# Histone Deacetylase SIRT1, Smooth Muscle Cell Function, and Vascular Diseases

**DOI:** 10.3389/fphar.2020.537519

**Published:** 2020-10-06

**Authors:** Fang Wang, Hou-Zao Chen

**Affiliations:** ^1^Department of Cardiology, China-Japan Friendship Hospital, Beijing, China; ^2^State Key Laboratory of Medical Molecular Biology, Department of Biochemistry and Molecular Biology, Institute of Basic Medical Sciences, Chinese Academy of Medical Sciences & Peking Union Medical College, Beijing, China

**Keywords:** SIRT1, vascular smooth muscle cells, vascular diseases, senescence, calorie restriction, SIRT1 activators

## Abstract

Vascular smooth muscle cells (VSMCs), located in the media of artery, play key roles in maintaining the normal vascular physiological functions. Abnormality in VSMCs is implicated in vascular diseases (VDs), including atherosclerosis, abdominal aortic aneurysm (AAA), aortic dissection, and hypertension by regulating the process of inflammation, phenotypic switching, and extracellular matrix degradation. Sirtuins (SIRTs), a family of proteins containing seven members (from SIRT1 to SIRT7) in mammals, function as NAD^+^-dependent histone deacetylases and ADP-ribosyltransferases. In recent decades, great attention has been paid to the cardiovascular protective effects of SIRTs, especially SIRT1, suggesting a new therapeutic target for the treatment of VDs. In this review, we introduce the basic functions of SIRT1 against VSMC senescence, and summarize the contribution of SIRT1 derived from VSMCs in VDs. Finally, the potential new strategies based on SIRT1 activation have also been discussed with an emphasis on SIRT1 activators and calorie restriction to improve the prognosis of VDs.

## Introduction

Cardiovascular diseases (CVDs) are the leading cause of death and disability all over the world. As mentioned in the “*2017 Cardiovascular Diseases Report in China*”, both the morbidity and mortality rates due to CVDs have increased in the past decades, with the current data reporting 270 million patients with hypertension, 11 million with coronary artery disease, 13 million with stroke, 5 million with pulmonary heart disease, and 4.5 million with heart failure. The occurrence and development of most CVDs are determined by vascular disorders, such as atherosclerosis, vascular neointimal formation, vascular stiffness, aortic aneurysm, and pulmonary hypertension; these are usually termed as vascular diseases (VDs) (Sakalihasan et al., 2005; Heusch et al., 2014; Luscher, 2018). Therefore, research on the mechanism underlying VDs is of great importance for the prevention of CVDs and the development of effective medical treatment strategies. Although the exact mechanism remains elusive, risk factors such as age, gender, diabetes mellitus (DM), dyslipidemia, cigarette smoking, and obesity contribute to the occurrence and development of VDs (Glass and Witztum, 2001; Isomaa et al., 2001; Ding et al., 2018).

Vascular smooth muscle cells (VSMCs) play a pivotal role in the maintenance of vascular homeostasis and the development of both the normal vasculature and VDs (Owens, 1995; Thompson et al., 1997; Intengan and Schiffrin, 2001). Under physiological conditions, VSMCs that exhibit the contractile phenotype are located in the media of vessels, whose functions include the regulation of vascular contraction, blood pressure, arterial tone-diameter, and flow distribution (Owens et al., 2004). Due to their remarkable differentiation plasticity in response to abnormal environmental stimuli, VSMCs tend to increase the rate of proliferation and migration as well as synthesize a variety of factors including pro-inflammatory chemokines and cytokines, matrix metalloproteinases (MMPs), growth factors, and reactive oxygen species (ROS) (Brozovich et al., 2016). This process of phenotypic switch is well-studied and known to be critically involved in the development of atherosclerosis, neointima formation, aortic aneurysm, and hypertension (Wang et al., 2018). Since aging is an independent risk factor for VDs, the role of cellular senescence has been remarkably focused on in the past few decades (Wang et al., 2013). VSMC senescence can be detected in aging vessels and is characterized by the decreased rate of proliferation and phenotypic switch mentioned above (Owens et al., 2004; Bennett et al., 2016). More importantly, senescence is not exactly the same as aging. For example, under certain environmental stressors, cells could exhibit senescence despite the organismal age (Rodier and Campisi, 2011). Although the current understanding of cellular senescence is not sufficient, it is reasonable to investigate the relationship between senescence-related molecules and progression of VDs in order to find new therapeutic approaches.

**Figure 1:**
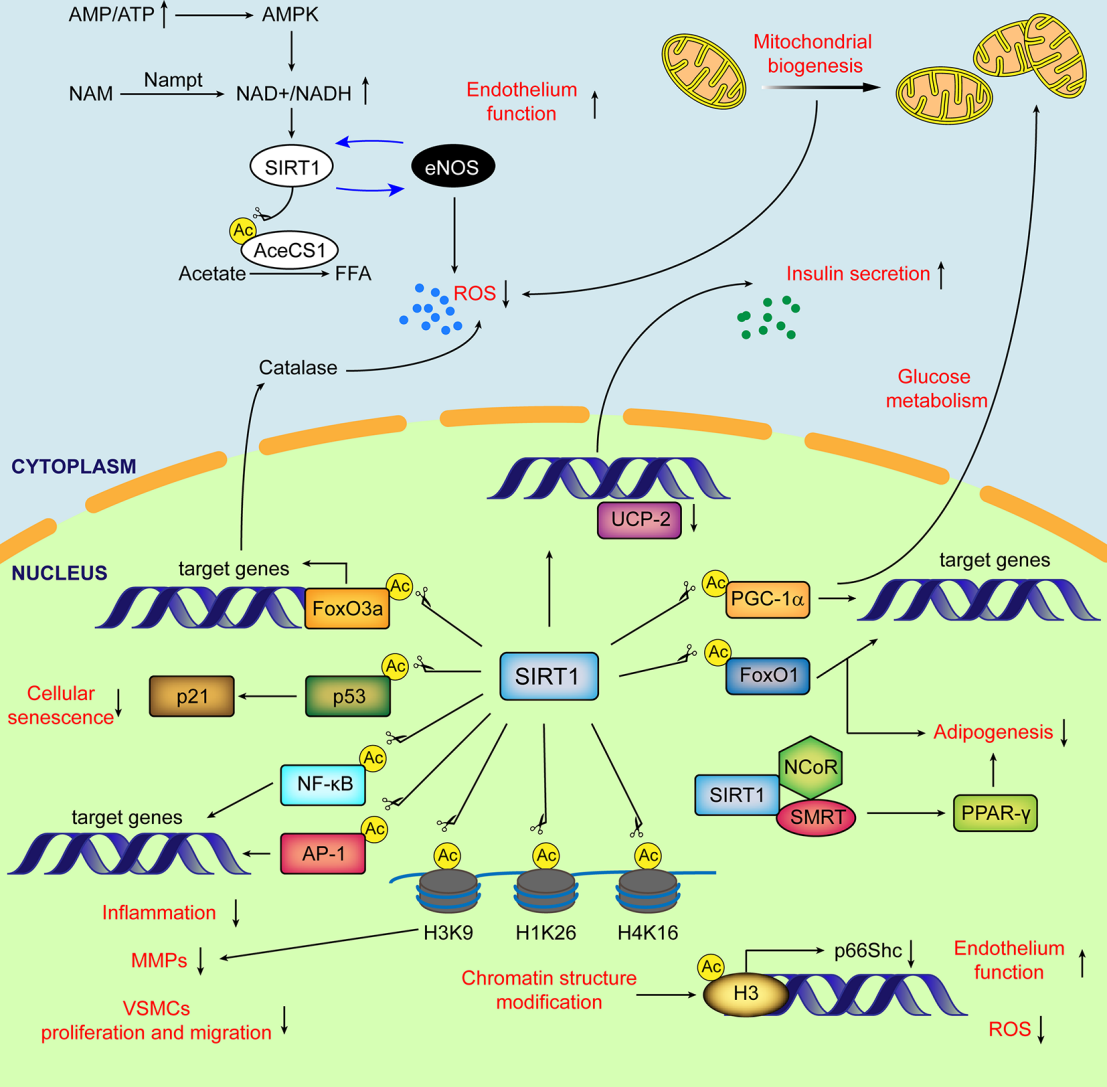
Schematic diagram of major subcellular functions of SIRT1 in mammals. SIRT1 is activated by elevated NAD^+^/NADH ratio. In cytoplasm, SIRT1 directly deacetylates AceCS1 to facilitate the synthesis of free fatty acid from acetate. The mutual promotion between SIRT1 and eNOS improves the endothelial function and decreases ROS production. SIRT1 stimulates transcriptional activity of PGC-1α, enhancing mitochondrial biogenesis and reducing the generation of ROS from mitochondria. In nucleus, by deacetylating histones and transcriptional factors, SIRT1 regulates glucose and lipid metabolism, cellular senescence, ECs and VSMCs function, inflammation, oxidative stress and extracellular matrix degradation. Ac, acetylation; AceCSs, Acetyl-CoA synthetases; AP-1, activator protein-1; eNOS, endothelial nitric oxide synthase; FFA, free fatty acids; FoxO, (Forkhead box class O); H3K9, histone H3 lysine 9; H4K16, histone H4 lysine 16; H1K26, histone H1 lysine26; MMPs, matrix metalloproteinases; NF-κB, nuclear factor-κB; NCoR, nuclear receptor co-repressor; PPARδ, peroxisome proliferator-activated receptor δ; PGC-1α, peroxisome proliferator-activated receptor δ co-activator 1α; ROS, reactive oxygen species; SMRT, silencing mediator of retinoid and thyroid hormone receptors; UCP, uncoupling protein.

The Sirtuin (SIRT) family of proteins, known as nicotinamide adenine dinucleotide (NAD)^+^-dependent protein deacetylases and ADP-ribosyltransferases, consist of seven members from SIRT1-SIRT7 (Finkel et al., 2009) (**Table 1**). SIRT1, the best-studied SIRT family member, has been demonstrated to regulate aging and age-related diseases (Bordone and Guarente, 2005; Alcendor et al., 2007; Yamamoto et al., 2007; Lavu et al., 2008) (Ding et al., 2018). The regulation of SIRT1 organized by the subcellular location (cytoplasm, nucleus, and mitochondrial biogenesis) in mammals as well as its functions is briefly listed in **Figure 1**. In recent years, the role of SIRT1 in vascular homeostasis and diseases in mammals has been well documented (Minamiyama et al., 2007; Lavu et al., 2008; Pillarisetti, 2008; Potente and Dimmeler, 2008). As most of the VDs are age-related, the regulation of vascular cell [especially endothelial cells (ECs) and VSMCs] senescence by SIRT1 has been deeply investigated (Kida and Goligorsky, 2016; Kitada et al., 2016). In this review, we summarize the existing knowledge regarding the role of VSMC-derived SIRT1 in cell senescence, and its role in the prevention and treatment of VDs. We suggest that vascular SIRT1, through its influence on a variety of pathophysiological processes including cellular senescence, can be a new potential therapeutic target that could be explored for its clinical relevance in the treatment of VDs.

**Table 1:** Main biological function of mammalian Sirtuins.

Sirtuin	Localization	Activity	Biological function	Target
SIRT1	Nucleus Cytoplasm	Deacetylase ADP-ribosyltransferase	Metabolism regulation, cell survival and aging, cellular senescence, DNA repair, cardiovascular protection, anti-oxidant, anti-inflammatory, regulation of apoptosis and autophagy	p53, FoxO1, FoxO4, NF-κB, histone H1, histone H3, histone H4, KU70, p300, PGC-1α, eNOS, AceCS1, E2F1, p73, NBS1, LXR
SIRT2	Cytoplasm	Deacetylase ADP-ribosyltransferase	Cell cycle, tumorgenesis	α-tubulin, histone H3 (K14), histone H4 (K16)
SIRT3	Mitochondria	Deacetylase ADP-ribosyltransferase	Cellular metabolism, regulation of apoptosis, cell survival and aging	AceCS2, GDH
SIRT4	Mitochondria	ADP-ribosyltransferase	Regulation of insulin secretion	GDH
SIRT5	Mitochondria	Deacetylase Demalonylase, Desuccinylase	Neurological regulation	CPS-1
SIRT6	Nucleus	Deacetylase ADP-ribosyltransferase	Metabolism regulation, cell survival and aging, DNA repair, cardiovascular protection	H3K9, H3K56, H3K18
SIRT7	Nucleus	Deacetylase	Cell survival and aging, RNA polymerase I activation	RNA polymerase I, p53

AceCSs, Acetyl-CoA synthetases; CPS1, carbamoyl phosphate synthetase 1; E2F1, E2F transcription factor 1; eNOS, endothelial nitric oxide synthase; FoxO, Forkhead box class O; GDH, glutamate dehydrogenase; H3K9, histone H3 lysine 9; H3K56, histone H3 lysine 56; H3K18, histone H3 lysine 18; LXR, liver X receptor; NBS-1, Nijmegen Breakage Syndrome-1; NF-κB, nuclear factor-κB; PGC1α, peroxisome proliferator-activated receptor-γ co-activator 1α.

## Influence of SIRT1 on Cellular Senescence in VSMCs

Cellular senescence represents a cell state triggered by numerous damaging stressors such as hypoxia, malnutrition, genotoxic stimuli, and oxidative stress. The main characteristics of cellular senescence include irreversible cell-cycle arrest, abnormal secretory phenotype, macromolecular (DNA, protein, and lipid) damage, and metabolic disorders (Gorgoulis et al., 2019). The workflow to recognize cellular senescence involves the screening of senescence-associated beta-galactosidase (SA-β-gal) activity and/or lipofuscin accumulation (SBB or GL13 staining). The second step is to stain the common markers (p16^INK4A^, p21^WAF1/Cip1^, and Lamin B1) and specific factors (PI3K/FOXO/mTOR, pro-inflammatory cytokines and chemokines, and MMPs) for the identification of cellular senescence. Cellular senescence comprises replicative senescence and stress-induced premature senescence (SIPS) (Kida and Goligorsky, 2016). Both types of VSMC senescence are implicated in the development of VDs (Gorenne et al., 2006; Kunieda et al., 2006; Matthews et al., 2006). The expression of endogenous SIRT1 in human VSMCs was markedly lower in old donors compared to that in young donors. The age-associated loss of SIRT1 expression resulted in the predisposition of VSMCs to cellular senescence with enhanced SA-β-gal positive staining, which is associated with the reduced cellular capacity of proliferation, migration, and UVB response. Even VSMCs from young donors showed cellular senescence after endogenous SIRT1 knockdown by siRNA. These findings indicate that SIRT1 downregulation contributes to cellular senescence in human VSMCs (Thompson et al., 2014).

Replicative senescence involves the cessation of cultured VSMC proliferation after a number of passages. Van der Veer et al. found that the overexpression of nicotinamide phosphoribosyltransferase (Nampt), a rate-limiting enzyme in the generation of NAD^+^ from nicotinamide, extended the lifespan of human VSMCs with the reduction of SA-β-gal positive VSMCs. This effect of Nampt is accompanied by increased SIRT1 deacetylase activity and p53 degradation, and is abrogated by the transduction of dominant-negative form of SIRT1 (Van Der Veer et al., 2007). Interestingly, only a little effect on the replicative longevity of human VSMCs was observed under the overexpression of SIRT1 alone. In contrast, the combined overexpression of SIRT1 and Nampt remarkably extended the lifespan of human VSMCs and attenuated the replicative senescence (Ho et al., 2009). Taken together, these findings indicate that Nampt plays an important role in the replicative longevity of human VSMCs that is dependent on the pronounced increase in SIRT1 activity.

It has been well documented that non-coding RNAs have a role in the regulation of VDs and cellular senescence (Ito et al., 2010; Congrains et al., 2012; Abdelmohsen et al., 2013; Boon et al., 2013; Bielak-Zmijewska et al., 2014). In the aortic VSMCs of aged mice, reduced microRNA-34a (miR-34a) expression was observed along with an enhanced p16 and p21 expression as well as repressed SIRT1 expression (well-known target gene of miR-34a) (Ito et al., 2010; Xu et al., 2012). In the cultured human aortic SMCs, decreased miR-34a level accompanied by SIRT1 down regulation was also observed during replicative senescence. More importantly, miR-34a overexpression along with SIRT1 depression could trigger cellular senescence even in young human aortic SMCs. Cellular senescence induced by miR-34a overexpression in VSMCs was rescued through exogenous SIRT1 protein transduction, indicating that the pro-senescent role of miR-34a is dependent on SIRT1 modulation (Badi et al., 2015). Long non-coding RNAs (lncRNAs), such as the antisense non-coding RNA in the INK4 locus (ANRIL), also participate in VSMC senescence. In aged VSMCs triggered by doubling passage, both ANRIL and SIRT1 were found to be downregulated, while the expression of microRNA-181a (miR-181a) was enhanced. Moreover, ANRIL overexpression significantly inhibited senescence and promoted the cell viability of VSMCs through the p53/p21 pathway repression. Mechanically, ANRIL overexpression directly reduced the expression of miR-181a. Meanwhile, miR-181a repressed SIRT1 expression by directly targeting the 3’UTR of SIRT1. In summary, non-coding RNAs participate in the replicative senescence of VSMCs through the modulation of SIRT1 expression (Tan et al., 2019).

Besides replicative senescence, SIRT1 is also involved in the SIPS of VSMCs. The renin–angiotensin system (RAS) is of vital importance in the occurrence and development of VDs (Ferder et al., 2002; Igase et al., 2005; Mehta and Griendling, 2007). Angiotensin II (Ang II), a pivotal molecule of the RAS, has been reported to induce premature senescence in both mice aorta and cultured VSMCs *via* the p53/p21 signal pathway (Daugherty et al., 2000; Kunieda et al., 2006; Minamino and Komuro, 2007). Therefore, in the subsequent studies, Ang II infusion was the most widely applied regime in the stimulation of vascular senescence. Our previous investigation showed that VSMC-specific SIRT1 overexpression ameliorated Ang II-induced premature senescence in mice aorta and cultured VSMCs, whereas VSMC-specific SIRT1 ablation exacerbated it. The anti-senescent function of SIRT1 was proved to be p21-dependent, which diminished NF-κB binding on the promoter of monocyte chemoattractant protein-1 (MCP-1), and blocked vascular inflammation (Chen et al., 2016). *Resveratrol (RSV), the best studied SIRT1 activator, is also known to be involved in vascular* senescence through the regulation of RAS. RSV was shown to significantly reduce the serum Ang II concentration as well as aortic prorenin receptor (PRR) and angiotensin converting enzyme (ACE) expression along with the increase in serum Ang-(1-7) level, the expression of ACE2, Ang II type 2 receptor (AT2R), and Mas receptor (MasR), which was accompanied by enhanced SIRT1 expression in aged mice as compared to that in the control group. As a result, vascular inflammation and oxidative stress were relieved after RSV treatment. In Ang II-infused VSMCs, RSV retarded premature senescence identified by SA-β-gal staining. The upregulation of AT2R and MasR was intensified under the administration of Ang II and RSV, in contrast to Ang II treatment alone (Kim et al., 2018). Taken together, VSMC-derived SIRT1 and its agonist, RSV, inhibits Ang II-primed premature senescence both *in vivo* and *in vitro*, which requires the involvement of the p53/p21 pathway.

Several studies also revealed that the regulation of VSMC senescence by certain agents is SIRT1-dependent. For instance, the activation of α7 nicotinic acetylcholine receptor (α7nAChR) by the selective agonist PNU-282987 blocked Ang II-induced premature senescence in cultured VSMCs and mice aorta. The anti-senescence effect of α7nAChR activation was mediated by increased SIRT1 deacetylase activity, which was abrogated after SIRT1 knockdown and inhibition by EX527 (Li et al., 2016). In addition, whey protein, a by-product of cheese manufacturing, has been demonstrated to inhibit Ang II-induced VSMC senescence, which promoted the mRNA and protein expression of SIRT1 in a dose- and time-dependent manner. Moreover, treatment with sirtinol, a SIRT1 inhibitor, compromised the anti-senescence effect of whey protein (Hwang et al., 2017). Research on the role of traditional Chinese medicine in cellular senescence has also gained significant attention. Buyang Huanwu decoction (BYHWD) is a traditional Chinese medicine formulation for the treatment of stroke. Serum containing BYHWD (BYHWS) attenuated Ang II-induced VSMC senescence. Furthermore, SIRT1 knockdown through siRNA abolished the protective role of BYHWS in VSMCs (Zhang et al., 2018).

**Figure 2:**
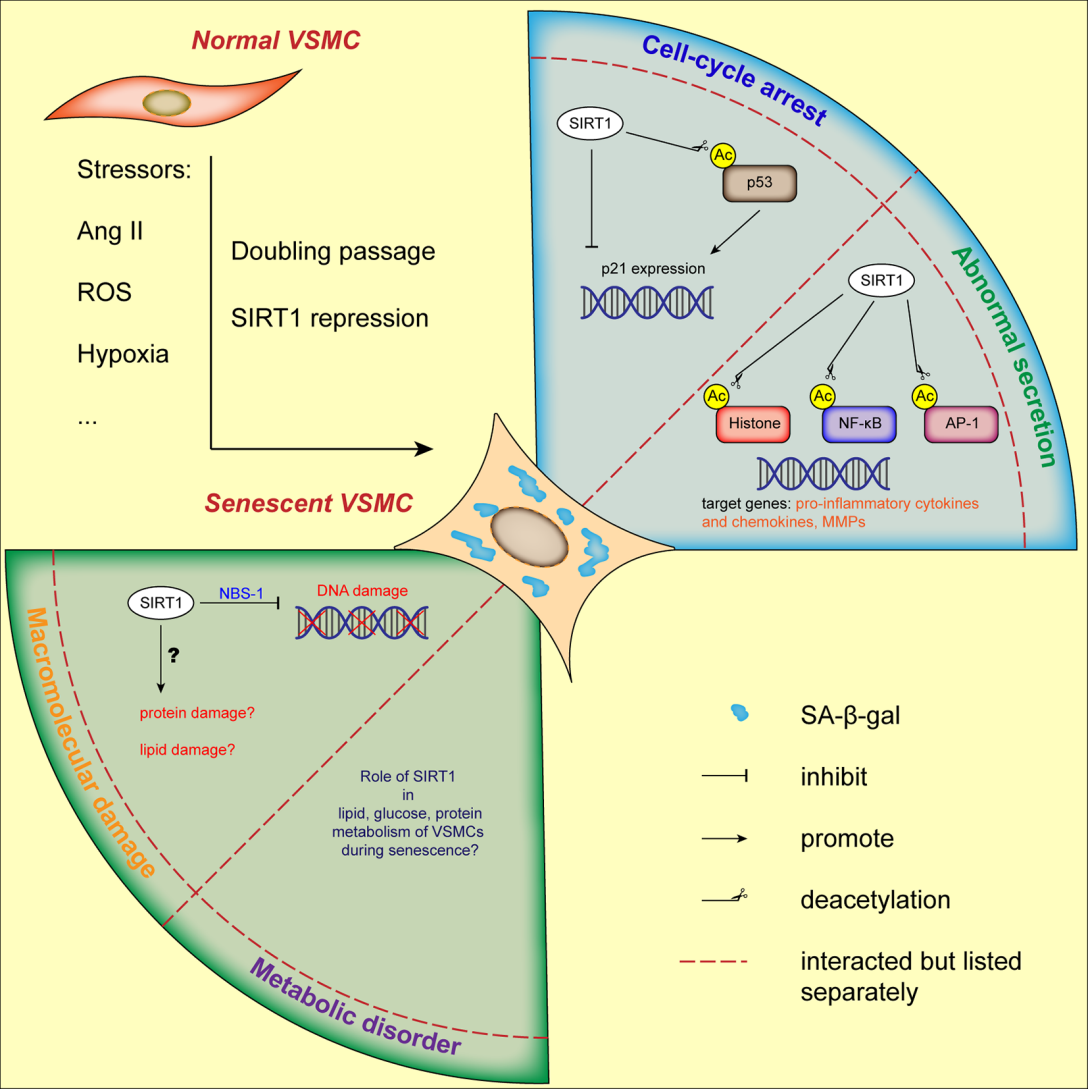
Current status of VSMCs senescence regulated by SIRT1. VSMCs senescence can be induced by stressors and doubling passage, characterized by augmented SA-β-gal activity and flattened morphology. Through its downstream molecules, SIRT1 is involved in cell-cycle arrest, abnormal secretory phenotype and DNA damage of VSMCs. The role of SIRT1 in other hallmarks of VSMCs senescence needs further elucidation. AP-1, activator protein-1; Ang II, angiotensin II; H3K9, histone H3 lysine 9; NBS-1, Nijmegen Breakage Syndrome-1; NF-κB, nuclear factor-κB; ROS, reactive oxygen species; VSMC, vascular smooth muscle cell.

In summary, we suggest that SIRT1 acts as a critical protective modulator involved in both replicative senescence and SIPS of VSMCs (**Figure 2**), which is mainly mediated by the repression of the p53/p21 pathway. It remains to be elucidated whether other characteristics of cellular senescence, such as macromolecular (protein and lipid) damage and metabolic disorders are involved in SIRT1-mediated VSMC function. It is worthy to note that the abnormal secretion of MMPs and pro-inflammatory molecules, unbalanced oxidative stress, deregulated capacity of VSMC proliferation and migration, and metabolic disorders in which VSMC-derived SIRT1 is implicated, participate not only in cellular senescence but also in the development of VDs.

## VSMC-Derived SIRT1 in VDs

### Atherosclerosis

Atherosclerosis is considered as the basic pathophysiological mechanism underlying coronary artery disease and ischemic cerebral VD. VSMCs are involved in almost all the stages of atherosclerosis, from the early neointima formation to the final plaque rupture. More importantly, the deregulated proliferation and migration as well as the stressor-induced phenotypic switch of VSMCs are deemed as pivotal processes in the development of atherosclerotic plaque (Basatemur et al., 2019).

In the primary stage of atherosclerosis, which is believed to be reversible, aberrant VSMC proliferation and migration are triggered by exposure to stressful insults, including ROS, pro-inflammatory cytokines, oxidized low-density lipoprotein (oxLDL), and pathological mechanical stretch. In cultured VSMCs, both oxLDL and hydrogen peroxide treatment could induce the pathological proliferation and migration, which is mediated by impaired SIRT1 expression (Hwang et al., 2016; Ma et al., 2016; Zhang et al., 2016). Consequently, the uncontrolled VSMC proliferation and migration contributes to neointima formation. However, it is noteworthy that the process of neointima formation is implicated not only in the early atherosclerotic plaque development but also the in-stent restenosis following percutaneous coronary intervention (PCI). In mouse model of neointima formation induced by carotid artery ligation or carotid artery wire injury, the vascular SIRT1 expression was found to be down regulated. Consistently, SMC-specific SIRT1 overexpression significantly inhibited neointima formation after vascular injury. Mechanically, in cultured VSMCs, SIRT1 overexpression remarkably ameliorated their proliferation and migration by transcriptionally decreasing the expression of cyclin D1 and MMP-9 through attenuating the acetylation levels of both c-Fos and c-Jun (Li et al., 2011). In addition, the role of interferon regulatory factor 9 (IRF9) in neointima formation is also dependent on VSMC-derived SIRT1. IRF9 global deficiency repressed the neointima formation in mice after carotid artery wire injury, whereas SMC-specific IRF9 overexpression resulted in its augmentation. According to the data from SIRT1/IRF9 double knockout mice, SIRT1-SMC-specific ablation abrogated the protective effect of IRF9 deficiency on neointima formation. The downstream mechanism underlying the function of SIRT1 involves the inhibition of VSMC proliferation and migration through the suppression of cyclin D1 and MMP-9 expression (Zhang et al., 2014). Taken together, the regulatory effect of SIRT1 on VSMC proliferation and migration relies on the cell cycle arrest at G1/S transition and the repression of MMPs, which potentiates SIRT1 as an intervention target for early atherosclerosis prevention.

The advanced stage of atherosclerosis is recognized by the presence of fibrous cap and necrotic core in the plaque. The plaque instability is considered as the pathological basis of acute coronary syndrome (ACS), which is characterized by a thin fibrous cap and plaque erosion leading to acute arterial narrowing or occlusion. The mRNA and protein levels of SIRT1 were markedly down regulated in human arterial atherosclerotic plaques compared to that in the normal vessels. In the aortae from *apoE^−/−^* mice fed with a high-fat diet, the aortic root plaque area and plaque vulnerability were aggravated under SMC-specific SIRT1 depletion along with enhanced DNA damage response activation and apoptosis. The effect of SIRT1 in DNA repair in VSMCs is mediated by the reduced activation of the repair protein Nijmegen Breakage Syndrome-1 but not p53 (Gorenne et al., 2013). Besides DNA damage and VSMC apoptosis, the imbalance between collagen synthesis and degradation within atherosclerotic plaques contributes to plaque instability resulting from the thinning of fibrous cap. The pro-inflammatory cytokine, IFN-γ, inhibited the transcription of collagen type I (*COL1A2*) gene encoding SMC-derived type I collagen through SIRT1 repression. SIRT1 activation relieved the inhibition of *COL1A2* transcription by reducing the acetylation of regulatory factor for X-box (RFX5), which binds to the promoter of *COL1A2* (Xia et al., 2012). MMPs are key stressors involved in collagen destruction within the atherosclerotic plaque. The production and activation of VSMC-derived MMP-2, MMP-1, and MMP-3 are respectively triggered by platelet activating factor (PAF) and oleic acid (OA), which are mediated by SIRT1 down regulation. Moreover, SIRT1 activation by RSV in VSMCs could inhibit PAF-stimulated MMP-2 expression through PAF receptor degradation (Kim et al., 2015; Chan et al., 2016). Another feature of late atherosclerosis is vascular calcification that intensifies the difficulty of PCI along with a greater possibility of coronary perforation and no-flow (Neumann et al., 2018). Vascular calcification is more likely to be detected in old CAD patients with diabetes and/or renal dysfunction. Consistent with the clinical findings, aortic medial calcification was accompanied by VSMC senescence in rat model of vascular calcification and renal failure induced by adenine-containing diet. In cultured VSMCs, inorganic phosphate (Pi) treatment markedly stimulated calcium deposit and cellular senescence with SIRT1 down regulation and increased acetylated (Ac)-p53 and p21 expression. Modulation of SIRT1 activity by RSV ameliorated Pi-induced senescence and calcification in VSMCs through the repression of p21 expression and osteoblastic phenotypic transition (Takemura et al., 2011). Another SIRT1 modulator, miR-34a, is also proved to be involved in vascular calcification. In the aortae and *in vitro* cultured VSMCs from miR-34a knockout mice, cellular senescence and calcification were remarkably relieved through the up regulation of SIRT1 (Badi et al., 2018). Thus, VSMC-derived SIRT1 is a critical modulator in the advanced stage of atherosclerosis, participating in plaque stability and vascular calcification.

In the past decades, the role of SIRT1, a longevity-related molecule, in atherosclerosis development has been widely studied not only in VSMCs but also in ECs (Ota et al., 2007; Potente et al., 2007; Zhang et al., 2008) and macrophages (Stein et al., 2010; Zhang et al., 2010; Stein and Matter, 2011), since aging is the independent risk factor for CAD. According to the data from studies in experimental animals, SIRT1 activators could be novel promising therapeutic options in both the early prevention and clinical therapy of arteriosclerotic cardiovascular diseases (ASCVDs), and need to be explored further in clinical trials for their relevance in a clinical setting.

### Abdominal Aortic Aneurysms (AAAs)

AAA formation is ranked as the 13^th^ leading cause of death in the United States, whose rupture accounts for the mortality rate of more than 80% (Sakalihasan et al., 2005). Very few patients with an AAA rupture could be rescued in the department of emergency (Tang et al., 2005). Moreover, most patients were reported to have died before arrival to the emergency room, thereby raising the importance of AAA prevention and elucidating its underlying mechanism. Observation from the aorta specimen of AAA donors and experimental animals revealed that VSMCs play pivotal roles in the pathogenesis of AAAs through regulating medial degradation, oxidative stress, and vascular inflammation (Thompson et al., 2002; Crawford et al., 2003). As advancing age is a major risk factor for AAA development (Brewster et al., 2003), research on age-related molecules such as SIRT1 could better our understanding on the pathogenesis and prevention of AAAs.

Compared with the adjacent control sections, the expression of SIRT1 was remarkably repressed in human AAA lesions. The *apoE^−/−^* mouse infused with Ang II is the most frequently used animal model to mimic AAAs in humans. After Ang II infusion for 4 weeks, both the incidence and the death rate were markedly reduced in SIRT1-VSMC–specific transgenic *apoE^−/−^* mice. Even without *apoE* knockout, an increase in both the prevalence and the mortality of AAAs was found in SIRT1-VSMC–deficient mice in contrast to those in the WT mice. Similar results were demonstrated in calcium chloride–induced mouse AAA model. The underlying mechanism involves the attenuation of vascular senescence by SIRT1 *via* the modulation of p53/p21 pathway (Chen et al., 2016). Due to its significance in the development of AAAs, the role of SIRT1-related life style change is discussed. Calorie restriction (CR), defined as reduced energy intake without malnutrition, is considered to be an important approach to retard aging and improve longevity (Dilova et al., 2007; Piper and Bartke, 2008). A number of studies demonstrated that the effects of CR are dependent on SIRT1 activation (Cohen et al., 2004; Guarente and Picard, 2005; Kaeberlein and Powers, 2007; Kume et al., 2010). After a 12-week regime of CR, the incidence of AAAs induced by Ang II infusion markedly decreased in *apoE^−/−^* mice compared to that in mice fed ad libitum, which was mediated by the epigenetic repression of MMP-2. However, the beneficial effect of CR on the development of AAAs was abolished in VSMC-specific SIRT1 knockout mice (Liu et al., 2016). Additionally, Licochalcone A (LA), a component derived from liquorice, attenuated Ang II-induced AAA formation in *apoE^−/−^* mice, which also required SIRT1 up regulation in VSMCs (Hou et al., 2019). Besides AAAs, SIRT1 is also important in the prevention of thoracic aortic aneurysm/dissection (TAAD). VSMC-specific SIRT1 knockout did not influence the incidence of TAAD but augmented the fatality rate in mice. In addition, VSMC-specific SIRT1 overexpression remarkably blocked TAAD development mainly through epigenetic downregulation of MMP-2 (Wang et al., 2020). Taken together, VSMC-derived SIRT1 is a promising intervention target for the prevention of life-threatening aortic diseases.

### Arterial Hypertension

Arterial hypertension is the most prevalent VD all over the world, whose morbidity in adults is around 30%–45%. Besides its instinctive danger, hypertension is considered a major risk factor for ASCVDs and aortic aneurysm/dissection. Unfortunately, the condition of blood pressure control is far from the ideal standard (Williams et al., 2018b). Although the exact mechanism underlying hypertension remains elusive, the vessel from hypertensive individuals exhibits aggravated arterial stiffness, vascular remodeling, oxidative stress, and vascular inflammation (Schulz et al., 2011; Luscher, 2018). Ang II of the RAS, a well-studied anti-hypertensive treatment target, triggers SMC constriction and hypertrophy as well as vascular remodeling by binding to angiotensin II type I receptor (AT1R) (Mehta and Griendling, 2007). Therefore, the modulation of Ang II signal pathway has been widely studied in the basic research of arterial hypertension and applied in clinical practice.

SIRT1 overexpression or activation by RSV was demonstrated to decrease the mRNA and protein expression of AT1R *in vitro*, suggesting the role of SIRT1 in blood pressure control (Miyazaki et al., 2008). Interestingly, SMC-specific SIRT1 ablation blocked the hypertensive response to Ang II, probably due to reduced expression of AT1R triggered by Ang II infusion (Fry et al., 2015). Moreover, after other vasoconstrictor such as phenylephrine stimulation, mice with SMC-specific SIRT1 ablation did not exhibit different blood pressure level compared with wild type mice (Fry et al., 2015). The *in vivo* experiments showed that SMC-specific SIRT1 overexpression could improve the systolic and diastolic blood pressure, arterial stiffness, and vascular remodeling in mice infused with Ang II (Gao et al., 2014; Fry et al., 2015). ROS production, vascular inflammation, and collagen synthesis in the vessel wall induced by Ang II were all inhibited by SIRT1 overexpression, which was attributed to the reduced transforming growth factor-β 1 (TGF-β1) expression *via* influencing the NF-κB binding on its promoter (Gao et al., 2014). In addition to Ang II, other stressors also contribute to high blood pressure and vascular remodeling. The deficiency of Klotho, predominantly expressed in the kidney and recognized as an aging-related molecule, could stimulate arterial stiffness and hypertension along with repressed aortic SIRT1 expression in mice (Gao et al., 2016). Through the reduction of AMP-activated protein kinase α (AMPKα) activity, Klotho loss-of-function led to enhanced vascular ROS production, medial collagen deposit, and elastin destruction in mice aorta, which could be blocked by SRT1720, a selective SIRT1 agonist. The expression of Klotho declines with advancing age partly elucidating the high prevalence of arterial hypertension in the elderly population (Gao et al., 2016). In light of the proven crosstalk between SIRT1 and AMPKα activity (Hou et al., 2008; Canto and Auwerx, 2009; Canto et al., 2009), SIRT1 activation can be a potential medical target to improve arterial stiffness and hypertension due to Klotho repression. Metabolic syndrome is also associated with the occurrence of hypertension as a large proportion of type 2 DM (T2DM) patients develop arterial hypertension (Williams et al., 2018b). In mice fed with a high-fat and high-sucrose (HFHS) diet, arterial stiffness measured by increased pulse wave velocity (PWV) was observed. Overnight fasting could ameliorate arterial stiffness induced by HFHS diet in WT mice but not in mice undergoing SMC-SIRT1 depletion. Furthermore, SMC-specific SIRT1 overexpression or SIRT1 activation by RSV significantly attenuated HFHS-diet-triggered arterial stiffness in mice along with reduced ROS production. Regulation of SIRT1 following the expression of oxidative-stress- and vascular-inflammation-related molecules was dependent on the decreased NF-κB binding activity (Fry et al., 2016). In summary, relying on the modulation of Ang II cascade, AMPKα activity, and NF-κB transcription activity, VSMC-derived SIRT1 exerts pivotal protective effects on blood pressure control and hypertension-related vascular remodeling and arterial stiffness, which may improve the long-term clinical prognosis in addition to the currently used anti-hypertensive drugs.

### Diabetic Vascular Dysfunction

DM is a major independent risk factor for coronary artery disease. Complications of DM such as diabetic vascular dysfunction predispose the individual to cardiovascular death (Isomaa et al., 2001; Nef et al., 2014). Main pathophysiological characteristics of diabetic vascular dysfunction include increased VSMCs proliferation and migration, vasoconstriction dysregulation, augmented inflammation and oxidative stress, and vascular calcification (Orimo et al., 2009). Diabetic vascular dysfunction also contributes to arterial stiffness, leading to the occurrence of hypertension (Scuteri et al., 2004; Williams et al., 2018a). At present, few strategies effectively retard the development of diabetic vascular dysfunction.

In both cultured VSMCs stimulated by high glucose and aortic VSMCs from diabetic rats induced by streptozotocin, SIRT1 expression was markedly depressed. However, tumor necrosis factor α (TNF-α) treatment did not induce SIRT1 downregulation in VSMCs (Toniolo et al., 2013). Activation of the endothelin-1 (ET-1) system is related to vasoconstriction dysregulation in diabetic conditions. It is probably due to the increased expression of endothelin type B (ET_B_) receptors in VSMCs under high glucose treatment, which is mediated by SIRT1 downregulation. RSV remarkably inhibited the ET_B_ receptor upregulation and vasoconstriction induced by high glucose treatment mainly through the repression of ERK1/2 phosphorylation (Lin et al., 2018). Metformin, a common used drug in DM management, also blocked homocysteine induced ET_B_ and ET_A_ receptor expression in VSMCs and vasoconstriction *in vivo*. These effects of Metformin were reverted by a SIRT1 specific inhibitor (Chen et al., 2020). VSMCs separated from *db/db* mice exhibited increased proliferation and migration, accompanied by enhanced miR-138 expression. Inhibition of miR-138 effectively attenuated VSMCs proliferation and migration through SIRT1 upregulation (Xu J. et al., 2015). VSMCs under high glucose treatment also showed accelerated calcification and cellular senescence. The calcification was mediated by the osteogenic diferentiation of VSMCs *via* RUNX2 signalling. It has been demonstrated that SIRT1 activation ameliorated, whereas SIRT1 inhibition promoted VSMC calcification through the regulation of RUNX2 pathway (Bartoli-Leonard et al., 2019). In summary, VSMC-dervied SIRT1 is critical in the prevention of diabetic vascular dysfunction, which could be a promising interventional target to improve the prognosis of DM patients. Taken together, research with experimental animals on the mechanism underlying SIRT1-mediated VDs has gained significant attention in recent times (**Table 2**), as it may lead to a breakthrough in the identification of a new potential therapeutic target for VDs; although the exact role of the anti-senescence effect of SIRT1 in the development of VDs need to be further investigated.

**Table 2:** Major role of SIRT1 in vascular diseases of experimental animals.

Function	Animal modification	Cell used in experiment	Mechanism	Targets	Representative references
Prevent atherosclerosis	EC-specific SIRT1-TG mice	HUVECs, SIRT1 overexpression by adenovirus transfection	Inhibit EC apoptosis and improve vasodilation	eNOS	Zhang et al. (2008)
	SMC-specific SIRT1-KO mice, heterozygotes or homozygotes	rat VSMCs, wild-type human SIRT1 or a deacetylation-deficient mutant SIRT1 was expressed by retrovirus-mediated gene transfer	Attenuate DNA damage and VSMC senescence	NBS-1	Gorenne et al. (2013)
	Global SIRT1^+/−^	murine RAW 264.7 cells (Mouse leukaemic monocyte macrophage cell line), *SIRT1^−/−^* mouse embryonic fibroblasts (MEF)	Reduce macrophage foam cell formation	Lox-1 and NF-κB	Stein et al. (2010)
Inhibit neointima formation	VSMC-specific SIRT1-TG/KO mice	rat VSMCs, SIRT1 overexpression or knockdown by adenovirus transfection; primary mouse VSMCs	Inhibit VSMC proliferation and migration and induce cell cycle arrest at G1/S transition	cyclin D1 and MMP-9 expression, c-Fos and c-Jun	Li et al. (2011); Zhang et al. (2014)
	Global SIRT1-TG mice	primary mouse VSMCs	Block the vascularization in neointima	HIF-1α	Bae et al. (2013)
Attenuate vascular calcification	SIRT1 knockdown in SMCs	primary human aortic SMCs, activation of SIRT1 by RSV, SIRT1 knockdown by small interfering RNA	Inhibit VSMC senescence and differentiation to osteoblast-like cells	p21	Takemura et al. (2011)
Block AAAs	VSMC-specific SIRT1-TG/KO mice	primary mouse VSMCs; human aortic SMCs, adenovirus-mediated knockdown of SIRT1	Attenuate VSMC senescence and inflammation	p21, NF-κB	Chen et al. (2016)
	CR; VSMC-specific SIRT1-KO mice	no *in vitro* data	Suppress Ang II-induced MMP-2 expression	histone H3K9	Liu et al. (2016)
Relieve arterial hypertension	VSMC-specific SIRT1-TG mice	no *in vitro* data	Relieve Ang II-induced vascular remodeling, oxidative stress and inflammation	TGF-β1, NF-κB	Gao et al. (2014)
	VSMC-specific SIRT1-TG/KO mice	primary mouse VSMCs; aortic tissue culture	Attenuate HFHS-induced arterial stiffness by blocking oxidative stress and inflammation	NF-κB	Fry et al. (2016)
Ameliorate diabetic vascular dysfunction	EC-specific SIRT1-TG mice	HUVECs, SIRT1 overexpression by adenovirus transfection	Improve EC function and reduce high glucose-induced oxidative stress through p66Shc downregualtion	histone H3	Zhou et al. (2011)
	EC-specific SIRT1-TG mice	HUVECs, SIRT1 overexpression by adenovirus transfection	Inhibit hyperglycemia induced vascular cell senescence	p53, p21, PAI-1 and MnSOD	Chen et al. (2012)
	EC-specific SIRT1-TG mice; RSV; PPARδ knockout mice	primary mouse aortic endothelial cells; aortic tissue culture; HUVECs, activation of SIRT1 by RSV	Promote endothelium-dependent vasodilation in diabetic and obese mice	PPARδ	Cheang et al. (2019)
	RSV; miR-138 inhibitor	VSMCs separated from *db/db* mice; SMC lines C-12511	Inhibit VSMC proliferation and migration	NF-κB	Xu et al. (2015)
	Metformin, Sprague-Dawley (SD) rats	primary rat VSMCs; aortic tissue culture	Blocke ET_B_ and ET_A_ receptor expression and vasoconstriction	ERK1/2, NF-κB	Lin et al. (2018) Chen et al. (2020)
		Primary human coronary artery VSMC	Inhibit calcification	RUNX2 pathway	Bartoli-Leonard et al. (2019)

AAA, abdominal aortic aneurysm; CR, calorie restriction; EC, endothelial cell; eNOS, endothelial nitric oxide synthase; HUVECs, Human umbilical vein endothelial cells; HIF-1α, hypoxia-inducible factor-1α; H3K9, histone H3 lysine 9; Lox-1, lectin-like oxLDL receptor-1; KO, knockout; MMP-9, matrix metalloproteinases 9; MnSOD, manganese superoxide dismutase; NBS-1, Nijmegen Breakage Syndrome-1; PAI-1, plasminogen activator inhibitor-1; PPARδ, peroxisome proliferator-activated receptor δ; RSV, resveratrol; TG, transgene; TGF-β1, transforming growth factor-β1; VSMC, vascular smooth muscle cell.

## Interventions Targeting SIRT1

Besides experimental animals, SIRT1 expression is also influenced in humans. For example, the myocardial SIRT1 expression was found to be lower in heart failure patients compared to that in healthy controls. Moreover, the treatment with statins, angiotensin receptor blockers, and metformin increased the mRNA level of SIRT1 in peripheral blood cells. More importantly, SIRT1 modulation was accompanied by attenuated oxidative stress, vascular inflammation, and cardiomyocyte apoptosis (Tabuchi et al., 2012; Lu et al., 2014; Xu W. et al., 2015; Turk Veselic et al., 2018; Janic et al., 2019). These consensus results raised the speculation that SIRT1 activation could be a promising intervention to improve the prognosis of CVDs. Current SIRT1-related interventions mainly include CR, RSV administration and NAD^+^ supplement, although the solid proof of their beneficial role in CVDs remains insufficient.

Early in 1996, a pilot study showed that coronary atherosclerotic lesions in 14 CAD patients was remarkably inhibited after 1-year CR. Subjects undergoing CR also exhibited lower serum cholesterol (Huh et al., 1996). Since obesity is a risk factor of CVDs, the direct potential effect of CR in the prevention of CVDs is attributed to body weight reduction. Another randomized controlled clinical trial indicated that weight loss due to CR significantly improves the cardiac ejection fraction and systolic output in CAD individuals (Oshakbayev et al., 2015). In 47 obese subjects undergoing 12-week CR, body weight reduction along with visceral fat area alteration was observed, which was correlated with relieved arterial stiffness measured by a decrease in cardio-ankle vascular index (CAVI) (Nagayama et al., 2013). Risk factors of CVDs are also affected by dietary changes. After a median 4-year follow-up, the traditional Mediterranean diet (MedDiet) involving high consumption of vegetables, grains, and olive oil; moderate consumption of fish and wine; and low consumption of red meat has been demonstrated to prevent the incidence of T2DM in a randomized trial enrolling 418 non-diabetic individuals (Salas-Salvado et al., 2011; Salas-Salvado et al., 2014). According to the data from several randomized clinical trials (RCTs) (Lefevre et al., 2009; Klempel et al., 2012; Weiss et al., 2016; Trepanowski et al., 2017), CR markedly lowered serum cholesterol, blood pressure, serum C-reactive protein, and ameliorated insulin resistance in both obese adults and healthy non-obese individuals. In summary, CR-mediated weight loss and metabolic benefit could act as a novel intervention to retard the progression of CVDs. Further large-scale studies are needed to determine the protective role of CR in specific CVDs along with elucidating the best acceptable and standard CR program.

RSV, a natural polyphenol, is abundant in grapes and red wine. In experimental animals, RSV was demonstrated to improve survival and retard the development of VDs (Howitz et al., 2003; Baur et al., 2006; Bass et al., 2007; Kaneko et al., 2011; Palmieri et al., 2011; Kim et al., 2018). Above all, a cross-sectional study that included 1,000 participants with a high risk of CVDs revealed that RSV intake is correlated with lower fasting serum glucose and triglycerides along with a lower heart rate. This large-scale study indicated the emerging role of RSV in reducing the risk of CVDs (Zamora-Ros et al., 2012). It has been proven that in humans either acute or long-term RSV consumption could improve vascular dilatation (Rakici et al., 2005; Wong et al., 2011; Wong et al., 2016). Similar to previous findings, in T2DM subjects, 12-week RSV supplementation ameliorated arterial stiffness, systolic blood pressure, and oxidative stress (Imamura et al., 2017). The role of RSV in ASCVDs is also discussed. In patients with carotid stenosis due to atherosclerotic plaque who received carotid endarterectomy, aterofsiol compound containing RSV was administrated 30 days before surgery. The carotid atherosclerotic plaque obtained from intervention individuals showed less lipid content (Amato et al., 2015). In stable CAD patients who underwent myocardial infarction, 3-month RSV supplementation significantly improved left ventricle diastolic function, endothelial function, and LDL-cholesterol level along with the inhibition of platelet aggregation (Magyar et al., 2012). Another RCT also demonstrated that subjects with stable angina pectoris who were treated with RSV combined with calcium fructoborate, showed repressed levels of serum C-reactive protein and N-terminal prohormone of brain natriuretic peptide, and less frequency of angina attack (Militaru et al., 2013). Taken together, in consensus with the findings from basic research, RCTs have suggested the beneficial effects of RSV supplementation in the management of CVDs, which is mediated by the regulation of vascular and cardiac function, lipid metabolism, oxidative stress, and inflammation.

As SIRT1 is a NAD^+^-dependent histone deacetylase, NAD^+^ supplement by providing its precursors such as nicotinamide riboside (NR) and nicotinamide mononucleotide (NMN) has been proved to be beneficial in treating CVDs and metabolic diseases in animal models (Bonkowski and Sinclair, 2016; Hershberger et al., 2017; Kane and Sinclair, 2018). In recent years, several phase I/II clinical studies are in process to determine the safety and efficacy of NR supplement. The results demonstrated that NR administration was well-tolerated in both healthy volunteers and obese individuals (Airhart et al., 2017; Martens et al., 2018). Unfortunately, findings of these clinical trials did not show the improved glucose metabolism and insulin sensitivity (Dollerup et al., 2018; Dollerup et al., 2019). The first human clinical study to evaluate the safety and the bioavailability of NMN began in 2016, although the results have not been reported yet (Kane and Sinclair, 2018). In the future, the assessment of blood pressure control, atherosclerotic plaque formation and arterial stiffness should be included in clinical trials of NAD^+^ supplement. Moreover, a greater number of subjects and longer follow-up time are also needed in following studies.

Due to the complexity of CR, NAD^+^ supplement and RSV treatment, some of their effects may not be mediated by SIRT1 activation. In clinical trials, it is difficult to determine whether the outcomes of CR, NAD^+^ supplement or RSV supplementation are dependent on SIRT1 activation. Therefore, selective SIRT1 activators, such as SRT2104, are being investigated in clinical trials. In healthy smokers receiving oral SRT2104 administration, serum lipid metabolic parameters including total cholesterol, LDL cholesterol, and triglyceride were found to be improved (Venkatasubramanian et al., 2013). SRT2104 supplementation also attenuated the level of serum pro-inflammatory cytokines and coagulation activation induced by intravenous injection with lipopolysaccharide in healthy subjects (Van Der Meer et al., 2015). It is necessary to conduct more large-scale RCTs to clarify the exact role of SIRT1 activation in the prognosis of CVDs. Current knowledge of common SIRT1 activators and inhibitors are summarized in **Table 3** (Alcain and Villalba, 2009a; Alcain and Villalba, 2009b; Dai et al., 2018).

**Table 3:** Summary of common SIRT1 activators and inhibitors.

SIRT1 activators	Information	Biological function
Resveratrol	Natural compound Not specific	Cardiovascular protection, neuroprotection, improved insulin sensitivity, increased aerobic capacity in muscle, anti-obesity
SRT1720/1460/2183	Synthetic SIRT1 activator	Preventing disorders such as diabetes, cancer, inflammation, cardiovascular diseases, neurodegenerative diseases.
SRT2104	Synthetic SIRT1 activator High specific	Improved lipid metabolism, anti-inflammation

**SIRT1 inhibitors**	**Information**	**Biological function**
Sirtinol	also inhibit SIRT2	Anticancer potential
Cambinol	also inhibit SIRT2	Anticancer potential, especially in Burkitt lymphoma
Ex-527	More specific	Possible to prevent Huntington’s Disease

## Conclusions and Perspectives

The development of VDs involves complicated pathophysiological processes affected by genetic and environmental regulation. Based on our current understanding of the underlying mechanism, the prevention and management strategies of VDs are far from satisfactory. Advancing age is considered a major risk factor in almost all VDs. Moreover, similar behaviors like enhanced cellular senescence, oxidative stress, vascular inflammation, activation of MMPs, and deregulated VSMC proliferation, migration and apoptosis are involved in vascular aging and the progression of VDs. Unlike skeletal muscle cells and cardiomyocytes, VSMCs are not terminally differentiated, and exhibit notable plasticity. Therefore, the phenotype of VSMCs varies according to the physiological cues and stressful insults, which may be complex but reversible. Research on aging-related molecules, such as SIRT1, in VSMCs will contribute to a better understanding of the pathogenesis of VDs so as to improve their prognosis and management.

Crosstalk between VSMCs, ECs, and macrophages ought to be discussed in subsequent works. In fact, they co-exist in the vasculature and are not isolated from each other. In a recently published study, authors revealed that VSMC-derived SIRT1 inhibited EC-genic angiogenesis after femoral artery ligation. The interaction was dependent on the delivery of exosome cZFP609 from VSMCs to ECs, leading to the repression of VEGF due to HIF-1α accumulation in the cytoplasm (Dou et al., 2020). Our previous findings also indicated that SA-β-gal-positive medial area after Ang II infusion was adjacent to the adventitia where infiltrated macrophages were mainly located. Therefore, SIRT1 in VSMCs may delay Ang II-induced vascular senescence by attenuating macrophage recruitment from adventitia through MCP-1 down-regulation (Chen et al., 2016). Additionally, macrophage-like foam cells located in atherosclerotic plaques are partly differentiated from VSMCs because of their plasticity (Basatemur et al., 2019). These data suggest that the cell fate and phenotype of ECs and macrophages may be determined by VSMC-derived SIRT1. Collectively, the effects of SIRT1 on the interaction between vascular cells appear to be more significant than its role in VSMCs alone. Hallmarks of the mutual relationships between vascular cells during senescence and the involvement of SIRT1 need to be identified in subsequent studies.

So far RCTs studying SIRT1 activation remain insufficient in clarifying its additional benefit on CVDs. In the future, above all, the significance of circulating SIRT1 in specific CVDs should be investigated. For example, the plasma SIRT1 level examined by ELISA was correlated with epicardial fat thickness in obese subjects (Mariani et al., 2016). Thus, circulating SIRT1 level could be a promising biomarker in predicting the clinical outcome of certain CVDs. Finally, further RCTs should be designed to determine whether SIRT1 selective agonists could influence solid clinical endpoints of CVDs, including the incidence of major adverse cardiovascular events (MACE) as well as death from vascular or other causes, hospitalization, and coronary revascularization.

## Author Contributions

FW was responsible for the literature search along with organizing and writing the manuscript. H-ZC was in charge of the architecture of figures and table, and revision of this article. All authors contributed to the article and approved the submitted version.

## Funding

This work was supported by grants from the National Natural Science Foundation of China (grant nos. 81700411 and 82030017), National Key Research and Development Project of China (2019YFA0801500), and Chinese Academy of Medical Sciences Innovation Fund for Medical Sciences (CIFMS2017-I2M-1-008, 2019-RC-HL-006).

## Conflict of Interest

The authors declare that the research was conducted in the absence of any commercial or financial relationships that could be construed as a potential conflict of interest.
